# Prevalence of Hypertension in a Community

**DOI:** 10.31729/jnma.5316

**Published:** 2020-12-31

**Authors:** Pradeep Paudel, Samir Chalise, Dinesh Raj Neupane, Narayan Adhikari, Shishir Paudel, Nim Bahadur Dangi

**Affiliations:** 1 Faculty of Health Sciences, School of Health and Allied Sciences, Pokhara University, Kaski, Nepal; 2 Department of Public Health, Manmohan Memorial Institute of Health Sciences, Kathmandu, Nepal

**Keywords:** *awareness*, *control*, *cross-sectional study*, *hypertension*, *prevalence*, *treatment*

## Abstract

**Introduction:**

Hypertension is one of the leading risk factors for global burden of disease and is of rising public health concerns in developing world including Nepal. However, few studies have focused on awareness, treatment, and control of hypertension among people living with this condition. In this scenario, this study aimed to find out the prevalence of hypertension and its awareness, treatment, and control among hypertensive patients residing in different parts of Kaski district, Nepal.

**Methods:**

A descriptive cross-sectional study was performed among 977 family members of 290 households from August to December 2017. Ethical approval was taken from the Institutional Review Committee (reference number: 73/074/75) of the Pokhara University Research Center. Simple random sampling was done. Hypertension screening was performed through averaging three values obtained by standardized aneroid sphygmomanometer in three observations. Primary data was collected through self-administered questionnaires and face-to-face interviews based on participant's preferences. Collected data were analyzed using Statistical Package for the Social Sciences version 20. Point estimate at 95% Confidence Interval was calculated along with frequency and proportion for binary data.

**Results:**

Of total 997 family members screened, 294 (29.49%) (26.66-32.32 at 95% Confidence Interval) had hypertension whereas only 127 (43.2%) were completely aware of their disease condition. 279 (94.9%) were taking antihypertensive medication and 201 (68.4%) had their blood pressure controlled.

**Conclusions:**

We found that almost one-fourth of the adult population in the community suffered from hypertension but less than half of the hypertensive patients are aware of their conditions.

## INTRODUCTION

Hypertension is a leading risk factor for the global burden of disease.^1,2^ In 2000 A.D. global estimate suggested approximately two-thirds of stroke and one-half of ischaemic heart disease were attributable to blood pressure.^3^ In 2005, this global burden was projected to rise from 0.9 billion in the year 2000 to 1.58 billion in 2025.^4^

World Health Organization estimates low- and middle-income countries account for two-thirds of hypertensive patients and it's of rising public health concerns in developing world including Nepal.^3,5^ In Nepal, a meta-analysis based on twenty-three studies reported the overall prevalence of hypertension at 28.4% (95% CI: 22.4-34.7%).^6^ With these rising concerns, it's important to understand the level of awareness, treatment, and control of hypertension to develop evidence-based strategies.^7^

In this scenario, this study aimed to seek out the prevalence of hypertension along with awareness, treatment, and control of hypertension among hypertensive patients of Kaski district, Nepal.

## METHODS

This was a descriptive cross-sectional study conducted among the residents of the Kaski District of Nepal from August to December 2017. The ethical approval was taken from the Institutional Review Committee (IRC) of Pokhara University Research Center (Ref No: 73/074/75). The people residing at Kaski districts at least for the past six months with an age above or equal to 18 years were included in the initial phase of the study for hypertension screening performed among 290 randomly selected households. However, residents suffering from severe physical and mental conditions limiting their ability to provide a proper response to the survey were excluded. The participants who were found to be hypertensive were eligible and thus involved in the study to understand their level of awareness, treatment and control of hypertension. Sample size was calculated as,

$$\begin{array}{ll} \text{n} & \text{= }{\text{Z}}^{\text{2}}\times \text{p}\times \text{(1}-\text{p)}/{\text{e}}^{\text{2}}\\ & \text{= }{\left(1.96\right)}^{\text{2}}\times 0.22\times 0.78/{(\text{0.05)}}^{\text{2}}\\ & \text{= }263.68\\ & \approx 264 \end{array}$$

where,

n = sample size
Z = 1.96 at 95% Confidence Interval (CI)
p = prevalence of hypertension among community of central Nepal, 22.4%^8^
q = 1-p
e = margin of error, 5%

Considering a 10% non-response rate the minimum sample size for this study was estimated at 290 hypertensive patients.

In this study, as there was no complete list of a hypertensive patient residing in the Kaski district so it was decided that the study will be carried out in two phases. In the first phase, the sample frame of the hypertensive patient was to be developed by screening hypertension among 290 randomly selected households representing each ward of the Metropolitan and four Rural-Municipalities of Kaski Districts. For this, the proportional to population size (PPS) method was adopted to estimate the required number of households to be selected from each ward of the study area to ensure representativeness based on the number of household data available from the 2011 census.^9^ In the second phase, after preparing the sampling frame, simple random sampling was to be used for random selection of a minimum of 290 hypertensive patients from the sampling frame. However, during our screening 294 hypertensive patients were identified, which was near to our sample size so all 294 hypertensive patients were selected.

Permission from the formal District Health Office was acquired prior to initiation of the study whereas informed consent from all the participants was acquired before the data collection. The confidentiality of the participants was assured and maintained through the coding of the participants. The voluntary participation of the subject was encouraged as the participants were notified about their option to withdraw from the study at any time on their will.

The screening of family members to identify hypertensive patients was performed by taking three blood pressure readings by using a standardized aneroid sphygmomanometer and averaging the value of three readings. The primary data for this study was collected using a self-administered questionnaire following the WHO Stepwise Survey Manual Approach.^10^ Based on the participant's preference, the questionnaire was distributed as a self-administered questionnaire to those participants who were willing to answer the question on their own, whereas participants who requested assistance were helped through a face-to-face interview approach. The questionnaire was divided into four sections. The first section consisted of a question regarding a participant's socio-demographic profile whereas the second section focused on their awareness of hypertension, its prevention, and consequences. The third section consisted of information regarding participant's hypertension treatment, management, and control status covering medicines underuse, participant's lifestyle, and their hypertension control status through blood pressure reading. For this, three blood pressure readings were made on the left arm with participants in a seated position after at least 5 minutes of rest and one-minute interval by a well-trained health care professional with a standardized aneroid sphygmomanometer. The means of three readings was used for the analysis. The fourth section consisted of participant's anthropometric measurements where the height was measured without shoes using a fixed measurement tape and body weight without heavy clothing was measured using a weight measurement device.

In this study, hypertension was defined as systolic blood pressure (SBP) of ≥140 mm Hg or diastolic blood pressure (DBP) of ≥90 mm Hg or those who were receiving anti hypertensive medication.^11^ Awareness was defined as a self-reported previous medical diagnosis of hypertension, its risk factors, and consequences. Treatment of hypertension was defined as the current use of antihypertensive medications intended to lower blood pressure. Body Mass Index was calculated as the weight in kilograms divided by square of height in meters (kg/m2) with a BMI cut-off point for overweight (25-29.9 kg/m2) and obesity(≥30 kg/m2) based on the categorization by WHO.^12^

The collected data was entered using Epidata software version 3.1 while the Statistical Package for the Social Sciences (SPSS) version 20 was used for the analysis. The collected data was analyzed using descriptive statistical methods such as frequency and percentage.

## RESULTS

Out of a total 290 randomly selected households for the screening of hypertension, we screened 997 household members and found that 294 (29.49%) (26.66-32.32 at 95% CI) of the members had hypertension. Among 997 participants 534 (53.5%) of the participants were females and the mean age (±SD) of the participants was 47.98 years ±17.427. Of the total participants, 793 (79.5%) participants were aged between 18 and 64 years whereas 204 (20.5%) of them were aged more than 64 years (Table 1).

**Table 1:** Socio-demographic profile of all the screened household members (n= 997).

Characteristics	Frequency n (%)
**Age group**	
Adult	793 (79.5)
Elderly	204 (20.5)
**Gender**	
Male	463 (46.4)
Female	534 (53.6)
**Marital status**	
Married	753 (75.5)
Unmarried	150 (15)
Widow	94 (9.5)
**Ethnicity**	
Brahmin	413 (41.4)
Chhetri	173 (17.4)
Dalit	88 (8.8)
Gurung	181 (18.2)
Others	142 (14.2)
**Educational status**	
Illiterate	409 (41)
Primary education	158 (15.8)
Secondary education	240 (24.1)
Higher education	190 (19.1)
**Occupational status**	
Employed in the Service Sector	260 (26.1)
Agriculture	286 (28.7)
Unemployed	308 (30.9)
Others	143 (14.3)

Of the total 294 hypertensive participants, the mean age of the participants was 61.94±12.83 years. 104 (35.4%) of the participants were Brahmins followed by Dalit 62 (21.1%) and other ethnicities. Similarly, 160 (55.4%) of participants were illiterate and 214 (77.3%) were engaged in a certain type of occupation.

The rate of smoking and alcohol consumption was noted among 79 (26.9%) and 61 (20.7%) of the hypertensive patients respectively whereas 129 (43.6%) were found to be overweight and 14 (16%) had obesity (Table 2).

**Table 2:** Distribution of demographic and health-related characteristics of hypertensive patients across gender (n= 294).

Variables	All hypertensives		
	Male (n= 117) n (%)	Female (n = 177) n (%)	Total n (%)
**Age**			
Adult	76 (65)	92 (52)	168 (57.1)
Elderly	41 (35)	85 (48)	126 (42.9)
**Marital status**			
Married	115 (98.3)	144 (81.4)	259 (88.1)
Unmarried	0 (0)	2 (1.1)	2 (0.7)
Widow	2 (1.7)	31 (17.5)	33 (11.2)
**Ethnicity**			
Brahmin	45 (38.5)	59 (33.3)	104 (35.4)
Chhetri	10 (8.5)	36 (20.3)	46 (15.6)
Gurung	18 (15.4)	21 (11.9)	39 (13.3)
Dalit	27 (23.1)	35 (19.8)	62 (21.1)
Others	17 (14.5)	26 (14.7)	43 (14.6)
**Occupation**			
Employed in Service Sector	39 (33.3)	21 (11.9)	60 (20.4)
Agriculture	37 (31.6)	69 (39)	106 (36.1)
Unemployed	18 (15.4)	62 (35)	80 (27.2)
Other	23 (19.1)	25 (14.1)	48 (16.3)
**Education**			
Illiterate	30 (25.6)	130 (73.4)	160 (54.4)
Primary education	26 (22.2)	21 (11.9)	47 (16)
Secondary education	41 (35)	19 (10.7)	60 (20.4)
Higher education	20 (17.1)	7 (4)	27 (9.2)
**Smoking**			
Smoker	47 (40.2)	32 (18.1)	79 (26.9)
Non-smoker	70 (59.8)	145 (81.9)	215 (73.1)
**Alcohol**			
Alcoholic	43 (36.8)	18 (10.2)	61 (20.7)
Non-alcoholic	74 (63.2)	158 (89.8)	233 (79.3)
**Exercise**			
Yes	87 (74.4)	104 (58.8)	191 (65)
No	30 (25.6)	73 (41.2)	103 (35)
**BMI categories**			
Underweight (<18.5 kg/m2)	5 (4.3)	7 (4)	12 (4.1)
Normal-weight (18.5-24.9 kg/m2)	44 (37.6)	64 (35)	106 (36.1)
Overweight (25.0-29.9 kg/m2)	50 (42.7)	79 (44.6)	129 (43.6)
Obese (≥30.0 kg/m2)	18 (15.4)	29 (16.4)	47 (16)
**Diabetes**			
Yes	21 (17.9)	24 (13.6)	45 (15.3)
No	96 (82.1)	153 (86.4)	249 (84.7)

Out of 294 hypertensive patients, 127 (43.2%) of the participants were found aware of their disease condition. Male participants were found to be more aware than female participants. Similarly, adult participants were more aware than elderly participants. Married participants and employed participants were found to be aware compared to their counterparts. Participants having higher education were found to be more aware than illiterate participants (Table 3).

**Table 3:** Socio-demographic characteristics with hypertensive awareness(n= 294).

Characteristics	All hypertensives		
	Aware n (%)	Not Aware n (%)	Total n (%)
Total	127 (43.2)	167 (56.8)	294 (100)
Age			
Adult	77 (45.8)	91 (54.2)	168 (57.1)
Elderly	50 (39.7)	76 (60.3)	126 (42.9)
Sex			
Male	63 (53.8)	54 (46.2)	117 (39.8)
Female	64 (36.2)	113 (63.8)	177 (60.2)
Marital Status			
Married	117 (45.2)	142 (54.8)	259 (88.0)
Unmarried	1 (50.0)	1 (50.0)	2 (0.01)
Widow	9 (27.3)	24 (72.7)	33 (11.99)
Ethnicity			
Brahmin	48 (46.2)	56 (53.8)	104 (35.4)
Chhetri	17 (37)	29 (63)	46 (15.6)
Gurung	15 (38.5)	24 (61.5)	39 (13.3)
Dalit	25 (40.3)	37 (59.7)	62 (21.0)
Others	22 (51.2)	21 (48.8)	43 (14.7)
Education			
Illiterate	45 (28.1)	115 (71.9)	160 (54.4)
Primary	20 (42.6)	27 (57.4)	47 (16.0)
Secondary	42 (70)	18 (30)	60 (20.4)
Higher Education	20 (74.1)	7 (25.9)	27 (9.2)
Occupation			
Employed in Service Sector	44 (73.3)	16 (26.7)	60 (20.4)
Agriculture	50 (47.2)	56 (52.8)	106 (36.0)
Unemployed	14 (17.5)	66 (82.5)	80 (27.2)
Others	19 (39.6)	29 (60.4)	48 (16.4)

**Figure 1:**
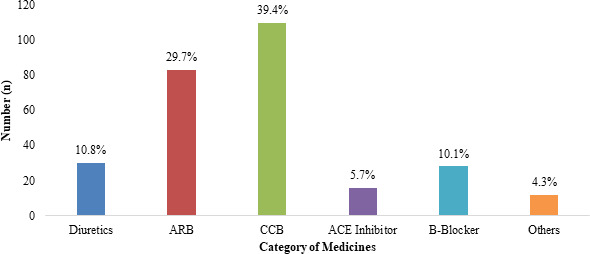
Category of AH medicine used by 279 hypertensive participants.

Among 294 hypertensive patients, 279 (94.9%) were taking antihypertensive (AH) medicines. It was found that Calcium Channel Blocker (CCB) was mostly prescribed medicine 109 (39.4%), followed by Angiotensin Receptor Blocker (ARB) 84 (29.7%), diuretics 30 (10.8%), ACE Inhibitor 16 (5.7%), and β-blocker 28 (10.1%) whereas only 12 (4.3%) of the participants were found to be taking other medicines. It was also noted that of these 279 hypertensive participants who were under treatment, the majority of then 181 (64.87%) were taking monotherapy (only single drug) in their medication regimen whereas 98 (35.13%) were using combined therapy (more than one drug) in their medication regimen. Similarly, 233 (83.6%) of the participants were taking medicines based on doctor's prescription while 46 (16.4%) took medicines directly from other healthcare professionals and pharmacists. (Figure 1).

More than two-thirds (68.4%) of hypertensive patients had their blood pressure under control after taking the medications (Table 4).

**Table 4:** Factors influencing hypertension control among patients who were under medications (n= 279).

Characteristics	All hypertensive patients on treatment		
	Controlled n (%)	Uncontrolled n (%)	Total n (%)
Total	201 (68.4)	78 (31.8)	279 (100)
Age			
Adult	116 (73.4)	42 (26.6)	158 (53.7)
Elderly	85 (70.2)	36 (29.8)	121 (46.3)
Gender			
Male	83 (74.1)	29 (25.9)	112 (40.1)
Female	118 (70.7)	49 (29.3)	167 (59.9)
Smoking			
Smoker	45 (61.6)	28 (38.4)	73 (26.16)
Non-smoker	156 (75.7)	50 (24.3)	206 (73.84)
Alcohol			
Alcoholic	37 (63.8)	21 (36.2)	58 (20.78)
Non alcoholic	164 (74.2)	57 (25.8)	221 (79.22)
Fat/oily food			
Oily	23 (67.6)	11 (32.4)	34 (12.18)
Normal	136 (69.7)	59 (30.3)	195 (69.89)
Less Oily	42 (84.0)	8 (16.0)	50 (17.93)
Exercise			
Yes	144 (77.0)	43 (23.0)	187 (67.02)
No	57 (62.0)	35 (38.0)	92 (32.94)
Awareness			
Aware	97 (80.08)	23 (19.2)	120 (43.02)
Not-aware	104 (65.4)	5 (34.6)	159 (56.98)
Diabetes			
Yes	24 (54.4)	20 (45.6)	44(15.8)
No	177 (75.3)	58 (24.7)	235(84.2)

## DISCUSSION

In this community-based study, it was noted that the prevalence of hypertension lies at 29.49%, with a higher proportion (79.5%) of the patient belonging to the 18 and 64 years. A similar prevalence rate of hypertension at 28% was reported by another community-based study performed in Lekhnath city of Western Nepal, which is one of the major city of Kaski district.^13^ Likewise, a study based on 1073 participants of Dhulikhel Municipality of Nepal found that 298 participants have hypertension yielding a similar prevalence rate of 27.02 percent.^7^ Similarly, a slightly higher rate of prevalence of 38.9% was noted in the communities of the mid-western region of Nepal.^14^ Furthermore, the prevalence rate noted in our study falls under the estimated prevalence of hypertension at 28.4% (95% CI: 22.4-34.7) reported by a systematic review and meta-analysis of 2019 which was based on twenty-three studies performed in Nepal.^6^

We observed that only 43.2% of the hypertensive patients were aware of their condition. Almost the same finding was noted in a study performed in the suburban town of Dhulikhel Municipality of Nepal where 43.6% of all hypertensive patients were aware of their hypertension status.^7^ In the same way, a study based on the suburban area of Kathmandu valley reported the rate of awareness to be at 41.1%.^15^ Similar observation was shared by a study conducted by Chow and colleagues among 142,042 participants of different communities of the different economies ranging from high-income countries to low-income countries where it was noted that only 26,877 (46.5%) of the participant were aware of their hypertension status.^16^ However, in the context of high-income countries, it was noted that a large proportion of the hypertensive patients were aware of their condition. As overall awareness rate of hypertension among the patients was noted to be at 54.33% in China,^17^ 74.4% in Brazil,^18^ 83% in Canada,^19^ and 88% in the US.^20^ This difference in the awareness level of different countries suggested that awareness can depend on the social, educational, and economic status of the country.

Out of the total of 294 Hypertensive participants of our study 279 were using antihypertensive (AH) medication yielding a treatment rate of 94.9%. This rate of treatment is remarkably higher than similar studies conducted in different parts of Nepal as only 29% hypertensive participants of Surkhet district, 31% hypertensive participants of Lekhnath city, and 76.1% hypertensive participants of Dhulikhel Municipality were reported to be on hypertension medication.^7,13,14^ These differences might be due to a higher level of awareness and relatively easier and affordable access to treatment for our study population due to the presence of social health insurance schemes, easy transport access, and other difference in the level of services and facilities.

In this study, it was also noted that 68.4% of the hypertensive patients had their blood pressure under control. Similarly, the study from Dhulikhel Municipality had also noted that only 35.3% of those who were on treatment had blood pressure under control. However, the controlled blood pressure was observed to be very low at 8.3% of the patients of Surkhet districts and 6% of the hypertensive patients in sub-urban area of Kathmandu valley.^14,15^ The prime reason behind this result could be a higher percentage of hypertensive patients were on antihypertensive medication and mostly Calcium Channel Blocker (CCB). The use of different medicine and combination of medicines might have drastically influenced the rate of blood pressure control among these studies. Further studies are needed to understand the effects of different drugs and combinations of drugs for the control of blood pressure in this population sub-groups. Further, improvement in health care services as compared to past, newly introduced health insurance schemes and the availability of low-cost drugs for hypertension management might also be the reason for these differences.

Although this study is one of the few studies to assess the awareness, treatment, and control status of hypertensive patients, this study has its limitations. The major limitation of this study is due to its descriptive nature over cross-sectional data which limits its scope as the causal inferences could not be drawn. Moreover, this study is based on one district of Nepal, this study is also not representative of the whole country due to the high ethnic, dietary, cultural, and geographical variation in the country.

## CONCLUSIONS

Through this study, it was noted that almost one-fourth of the adult population suffers from hypertension but less than half of the hypertensive patients are aware of their conditions. A statistical relationship exists between awareness of hypertension and its control. Therefore, proper medical services and counseling should be provided by the doctor and other healthcare professionals to increase the level of awareness, treatment, and control of hypertension.
